# Site-1 Protease-Derived Soluble (Pro)Renin Receptor Contributes to Angiotensin II–Induced Hypertension in Mice

**DOI:** 10.1161/HYPERTENSIONAHA.120.15100

**Published:** 2020-12-07

**Authors:** Ye Feng, Kexin Peng, Renfei Luo, Fei Wang, Tianxin Yang

**Affiliations:** From the Department of Internal Medicine, University of Utah and Veterans Affairs Medical Center, Salt Lake City.

**Keywords:** amiloride, angiotensin II, blood pressure, epithelial sodium channels, renin

## Abstract

Activation of PRR ([pro]renin receptor) contributes to enhancement of intrarenal RAS and renal medullary α-ENaC and thus elevated blood pressure during Ang II (angiotensin II) infusion. The goal of the present study was to test whether such action of PRR was mediated by sPRR (soluble PRR), generated by S1P (site-1 protease), a newly identified PRR cleavage protease. F1 B6129SF1/J mice were infused for 6 days with control or Ang II at 300 ng/kg per day alone or in combination with S1P inhibitor PF-429242 (PF), and blood pressure was monitored by radiotelemetry. S1P inhibition significantly attenuated Ang II–induced hypertension accompanied with suppressed urinary and renal medullary renin levels and expression of renal medullary but not renal cortical α-ENaC expression. The effects of S1P inhibition were all reversed by supplement with histidine-tagged sPRR termed as sPRR-His. Ussing chamber technique was performed to determine amiloride-sensitive short-circuit current, an index of ENaC activity in confluent mouse cortical collecting duct cell line cells exposed for 24 hours to Ang II, Ang II + PF, or Ang II + PF + sPRR-His. Ang II–induced ENaC activity was blocked by PF, which was reversed by sPRR-His. Together, these results support that S1P-derived sPRR mediates Ang II–induced hypertension through enhancement of intrarenal renin level and activation of ENaC.

## Abstract 2

Supplemental Digital Content is available in the text.

**See Editorial, pp 417–419**

In 2002, Nguyen et al^1^ cloned a specific transmembrane receptor for prorenin and renin, termed PRR ([pro]renin receptor). At structural level, PRR is also an accessory protein (M8-9) of the vacuolar-type H^+^-ATPase and designated ATP6AP2.^2^ PRR is composed of a large N-terminal extracellular domain, a single transmembrane domain, and a short cytoplasmic domain.^3^ The extracellular domain is cleaved by protease to generate a sPRR (soluble form of PRR) which is detected in biological fluids.^4^ Although furin and ADAM19 were initially shown to be the cleavage proteases, recent reports from Nakagawa et al^5^ and us^6^ consistently show that S1P (site-1 protease) serves as a predominant source of sPRR both in vitro and in vivo.

**See Editorial pp 417–419**

Within the kidney, PRR is abundantly expressed in intercalated cells of the CD albeit with relatively lower expression in multiple nephron structures.^7^ PRR has been implicated to play an essential role in regulation of a spectrum of renal functions under various physiopathological processes, particularly renal control of blood pressure (BP) during Ang II (angiotensin II)–induced hypertension.^8,9^ Increasing evidence suggests that Ang II–induced hypertension relies on prostaglandin-dependent activation intrarenal RAS and PRR.^8–14^ However, the detailed mechanism of how PRR is involved in BP regulation remains elusive.

Emerging evidence reveals biological functions of sPRR in renal handling of Na^+^ and water. In this regard, sPRR was originally shown to directly upregulate AQP2 (aquaporin-2) expression via activation of β-catenin signaling, thus contributing enhancement of urine concentrating capability.^15^ Subsequently, S1P-derived sPRR was found to target both AQP2 and V2R (vasopressin type 2 receptor) to effectively influence water reabsorption and [Arg8]-Vasopressin (AVP) sensitivity. However, there is no prior study to examine the potential role of S1P-derived sPRR during Ang II–induced hypertension.

## Methods

The authors declare that all supporting data are available within the article (and its Data Supplement).

### Animals

Male 10- to 12-week-old B6129SF1/J mice were purchased from the Jackson Laboratory. They are F1 hybrid offspring of a cross between C57BL/6J females (B6) and 129S1/SvImJ males (129S). All animals were cage-housed and maintained in a temperature-controlled room with a 12:12-hour light-dark cycle, with free access to tap water and standard mouse chow for 14 days. The present animal studies were approved by the Animal Care and Use Committee at the University of Utah.

### Mouse Experiments

B6129SF1/J mice were infused for 6 days with control, Ang II (Ang II at 300 ng/kg per day), Ang II + 4-[(Diethylamino)methyl]-N-[2-(2-methoxyphenyl)ethyl]-N-(3R)-3-pyrrolidinyl-benzamide dihydrochloride (PF) (PF at 20 mg/kg per day; HY-13447A, Medchem Express) via a subcutaneously implanted minipump (Alzet model 1007D, Alza) or in conjunction with intravenous sPRR-His infusion at 30 μg/kg per day via jugular vein catheterization connected to a separate osmotic minipump as previously reported.^15^ The same doses of Ang II, PF, and sPRR-His have been validated by our previous studies.^15,16^ The radiotelemetric device was implanted via catheterization of carotid artery and was turned on for 4 hours per day from 5:00 pm to 9:00 pm. At the end of the experiment, under general anesthesia, urine was collected from puncturing the bladder, blood was withdrawn by puncturing vena cava, and the kidney was harvested and cut into cortex and the inner medulla and snaps frozen. Twenty-four hours urine collection was performed on separate groups of the animals receiving the same treatments except that they were not instrumented with radiotelemetric devices.

### Renin Activity Assay

Renin activity assay was performed as previously described.^9^ Briefly, renin activity in urine and renal inner medulla was determined by the delta value of the Ang I generation using an ELISA kit from the sample incubating at 4 °C and 37 °C for 1 hour, respectively. Total renin content was measured with excessive angiotensinogen plus trypsinization and active renin content with excessive angiotensinogen. Urine and tissue samples were spiked with 1 µmol/L synthetic renin substrate tetradecapeptide (R8129; Sigma-Aldrich, St Louis, MO). After incubation at 37 °C for 18 hours, Ang I generation was assayed by using an Ang I enzyme immunoassay kit (S-1188; Peninsula Laboratories International, SanCarlos, CA), according to the manufacturer’s instructions. The values were expressed as nanograms per milliliter per hour of generated Ang I. For measurement of total renin content, trypsinization was performed to activate prorenin to renin.^17^ The samples were incubated with trypsin derived from bovine pancreas (100 g/L, T1426, Sigma-Aldrich) in 37 °C for 18 hours. The reaction was then terminated with soybean trypsin inhibitor (100 g/L, T6522; Sigma-Aldrich) at 37 °C for 1 hour. Renin activity was determined in the native condition, active renin content with excessive angiotensinogen, and total renin content with excessive angiotensinogen plus trypsinization.

### Immunoblotting

Renal tissues, including the cortex and the inner medulla, were lysed and subsequently sonicated in phosphate buffered saline (PBS) that contained 1% Triton x-100, 250 μmol/L phenylmethanesulfonyl fluoride, 2 mmol/L ethylenediaminetetraacetic acid (EDTA), and 5 mmol/L dithiothreitol (pH 7.5). Protein concentrations were determined by the use of coomassie reagent. Forty micrograms of protein for each sample were denatured in boiling water for 10 minutes, then separated by SDS-PAGE, and transferred onto nitrocellulose membranes. The blots were blocked overnight with 5% nonfat dry milk in Tris-buffered saline, followed by incubation for overnight with primary antibody. After being washed with Tris-buffered saline, blots were incubated with horseradish peroxidase-conjugated secondary antibody and visualized using Enhanced Chemiluminescence. The blots were quantitated by using Imagepro-plus. Primary antibodies are as follows: rabbit anti-PRR antibody (catalog no. HPA003156; Sigma), rabbit anti-α-epithelial sodium channel (ENaC) antibody (catalog no. SPC-403D; Stressmarq Biosciences), mouse anti-β-actin antibody (catalog no. A1978; Sigma).

### Quantitative Real-Time Polymerase Chain Reaction

Total RNA isolation and reverse transcription were performed as previously described.^9^ Oligonucleotides were designed using Primer3 software (available at http://frodo.wi.mit.edu/cgi-bin/primer3/primer3_www.cgi). Primers were as follows: for α-ENaC: 5′-gcgacaacaatccccaag-3′ (sense) and 5′-tgaagcgacaggtgaagatg-3′ (antisense); for β-ENaC: 5′-aagcacctgtaatgcccaag-3′ (sense) and 5′-atagcccatccccaccag-3′ (antisense); for γ-ENaC: 5′-cgaagaaactggtgggattt-3′ (sense) and 5′-gatggtggaaaagcgtgaag-3′ (antisense); for Renin: 5′-gatcaccatgaagggggtctctgt-3′ (sense) and 5′-gttcctgaagggattcttttgcac-3′ (antisense); for GAPDH: 5′-gtcttcactaccatggagaagg-3′ (sense) and 5′-tcatggatgaccttggccag-3′ (antisense); and for Angiotensinogen: 5′-tatccactgacccagttcttt-3′ (sense) and 5′-aagtgaacgtaggtgttgaaa-3′ (antisense).

### Cell Culture Experiments

The mouse cortical collecting duct cell line (mpkCCD) was established from a transgenic mouse expressing SV40 large T antigen under the control of the SV40 enhancer/L-type pyruvate kinase promoter.^18^ mpkCCD cells were grown in Transwells (catalog no. 29442-074, VWR) with Dulbecco's Modified Eagle Medium (DMEM)/F-12 medium containing 10% fetal bovine serum (FBS) (Thermo Fisher Scientific), 0.5 μmol/L 8-Br-cAMP (MilliporeSigma), 130 mmol/L NaCl (Mallinckrodt Chemical), and 80 mmol/L urea (Mallinckrodt Chemical). The cells were pretreated with Aliskiren (1 μmol/L; catalog no. HY-12176, MCE) for 0.5 hours or PF (10 μmol/L) for 1 hour or transfected with S1P siRNA or the corresponding scrambled siRNA for 48 hours, and then treated with Ang II (1 μmol/L) or sPRR-His (10 nmol/L) for 24 hours. At the end of the experiments, the medium was collected for biochemical assays.

### Electrophysiological Measurements of Transepithelial Na^+^ Transport

Electrophysiology experiments were performed on immortalized mpkCCD cells after the cell monolayers reached confluence. Transepithelial Na^+^ transport was recorded by using the Ussing chamber device (Physiological Instruments, San Diego, CA).^19^ In performing the Ussing chamber technique, the voltage was clamped at zero using a VCC600 voltage-clamp apparatus (Physiological Instruments), and then the short-circuit current was recorded using Ag-AgCl electrode in agar brides. Positive short-circuit current reflects the active transport of cation (Na^+^) from apical side to basolateral side of media or transport of anion (Cl^−^) from basolateral to apical side of media. For both measurements, amiloride-sensitive component was taken as an index of ENaNaC activity. To examine the role of S1P-derived sPRR in regulation of Ang II–induced ENaC activation, the cells were pretreated with PF (10 μmol/L) for 1 hour or transfected with S1P siRNA or the corresponding scrambled siRNA for 48 hours, and then treated for 24 hours with Ang II (1 μmol/L) or sPRR-His (10 nmol/L). To explore the role of renin in regulation of Ang II–induced ENaC activation, the cells were pretreated with Aliskiren (1 μmol/L) for 0.5 hours and then treated with Ang II for 24 hours.

### Enzyme Immunoassay

sPRR in urine and biological fluids were determined by using the following commercially available enzyme immunoassay kits according to the manufacturer’s instructions: the kit for sPRR (catalog no. JP27782, IBL).

### Small Interference RNA Transfection

S1P siRNA oligonucleotides were purchased from Invitrogen (catalog no. 4390771). At 50% to 70% confluence, the mpkCCD cells were transfected with siRNA or nontargeting scrambled siRNA using Lipofectamine RNAiMAX Transfection Reagent (Invitrogen, catalog no. 13778-030).

### Statistics

Data are summarized as means ± SEM. All data points represent animals that were included in the statistical analyses. Sample sizes were determined on the basis of similar previous studies or pilot experiments. Statistical analysis for animal experiments was performed by using ANOVA with the Bonferroni test for multiple comparisons or by paired or unpaired Student *t* test for 2 comparisons. The Student *t* tests were performed with 2-tailed *t* test. The *P*<0.05 was considered statistically significant.

## Results

### Radiotelemetry Monitoring of BP

**Figure 1.**
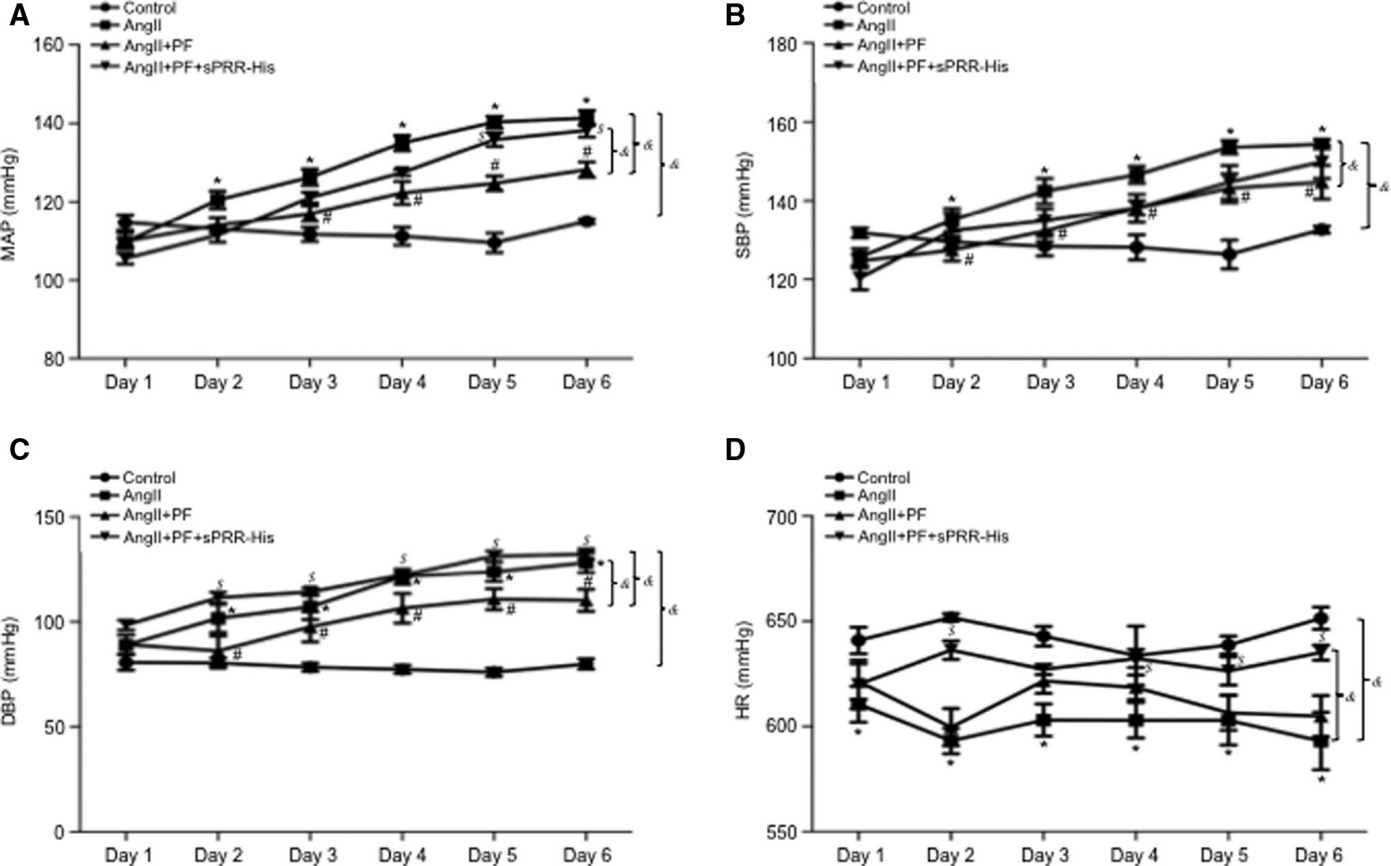
**Effect of S1P (site-1 protease) inhibition on Ang II (angiotensin II)–induced hypertension in mice.** B6129SF1/J mice were randomly divided into the following 4 groups: (1) Control (CTR), (2) Ang II, (3) Ang II + PF, or (4) Ang II + PF + sPRR (soluble [pro]renin receptor)-His. Radiotelemetry was performed to monitor mean arterial blood pressure (MAP; **A**), systolic blood pressure (SBP; **B**), diastolic blood pressure (DBP; **C**), and heart rate (HR; **D**) over 6 d. &*P*<0.05, the interaction detected by repeated-measures ANOVA. *P<0.05 vs CTR for the corresponding period (Bonferroni test). #*P*<0.05 vs Ang II for the corresponding period (Bonferroni test). $*P*<0.05 vs Ang II + PF for the corresponding period (Bonferroni test). Data are mean ± SE. N=4–8 mice per group.

In light of recent discovery of S1P/sPRR pathway in the kidney,^6,15^ the present study attempted to test the involvement of this pathway in a mouse model of Ang II–induced hypertension. The study was designed to test whether S1P inhibition with PF attenuated Ang II–induced hypertension and if so further to examine whether supplement of an exogenous sPRR was able to reverse the antihypertensive action of PF. For the first series of experiments, male 10- to 12-week-old B6129SF1/J mice were randomly divided into the following groups: (1) control, (2) Ang II, (3) Ang II + PF, and (4) Ang II + PF + sPRR-His. Radiotelemetry was conducted to monitor daily changes in mean arterial pressure (MAP), systolic BP (SBP), diastolic BP (DBP), and heart rate. As compared with control group, Ang II infusion over 6 days at a pressor dose of 300 ng/kg per day gradually and significantly elevated MAP, SBP, and DBP (day 6: delta MAP 26.4±3.2 mm Hg; delta SBP 21.8±1.9 mm Hg; and delta DBP 48.8±9.4 mm Hg). Likely, due to baroreflex response, heart rate was decreased immediately following Ang II infusion (Figure 1D). In contrast, the increases in Ang II + PF group were less for MAP and SBP and were completely blocked for DBP as compared with Ang II group. Interestingly, heart rate in Ang II + PF group exhibited a similar drop at the beginning, which remained suppressed over the rest of the experimental period (Figure 1D). Addition of sPRR-His infusion did not affect BPs within the first 4 days but significantly restored the hypertensive response in PF treated mice as reflected by the changes in MAP, SBP, and DBP (Figure 1A through 1D). Body weight was determined on the last day of the experiment and neither of these parameters showed significant difference among the 4 groups (Figure S1 in the Data Supplement).

### Analysis of Renin Parameters

**Figure 2.**
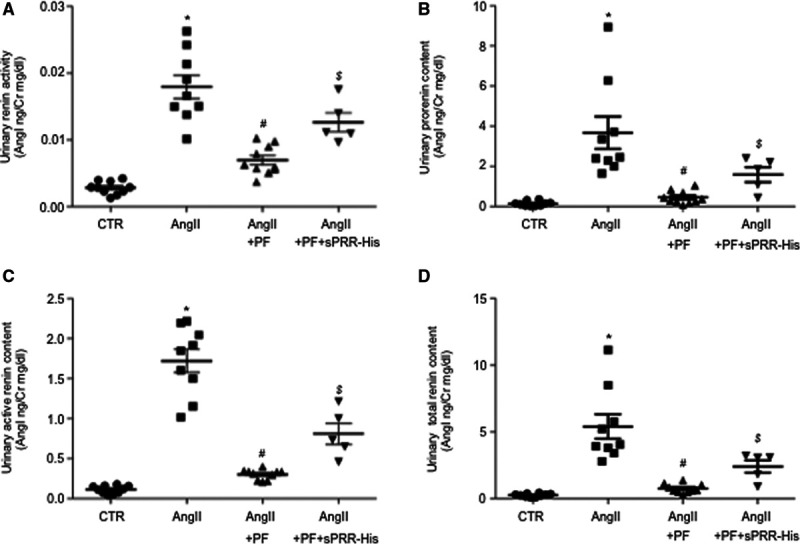
**Assessment of urinary renin levels in Ang II (angiotensin II) infused mice during S1P (site-1 protease) inhibition alone or supplemented with sPRR (soluble [pro]renin receptor)-His.** B6129SF1/J mice were randomly divided into the following 4 groups: (1) Control (CTR), (2) Ang II, (3) Ang II + PF, and (4) Ang II + PF + sPRR-His. Urine samples were assayed for renin activity (**A**), prorenin content (**B**), active renin content (**C**), and total renin concentration (**D**). The values were normalized by creatinine. Data are mean ± SE. N=5–10 mice per group. *P<0.05 vs CTR. #*P*<0.05 vs Ang II. $*P*<0.05 vs Ang II + PF.

During Ang II infusion, circulating renin is suppressed but urinary renin is enhanced, highlighting the distinct responses of intrarenal and systemic origins of renin.^11,12,14,20,21^ Overactivation of intrarenal renin-angiotensin-system (RAS) contributes to pathogenesis of hypertension induced by Ang II infusion^22–24^ or inhibition of nitric oxide synthesis.^25^ We, therefore, determined urinary renin activity, active renin content, prorenin content, and total renin content at day 6 of treatment with control, Ang II, Ang II + PF, or Ang II + PF + sPRR-His. The values were normalized by creatinine. As expected, all renin parameters from the urine samples were remarkably increased by Ang II infusion (Figure 2). Among these renin parameters, the increased urinary renin activity was suppressed by ≈50%, and the response of rest of the renin parameters, including urinary prorenin content, renin content, and total renin content, were all nearly completely abolished by PF. Addition of sPRR-His was able to partially restore the response of all renin parameters to Ang II (Figure 2). These results suggest that S1P-derived sPRR plays an important role in determining intrarenal renin response to Ang II infusion.

**Figure 3.**
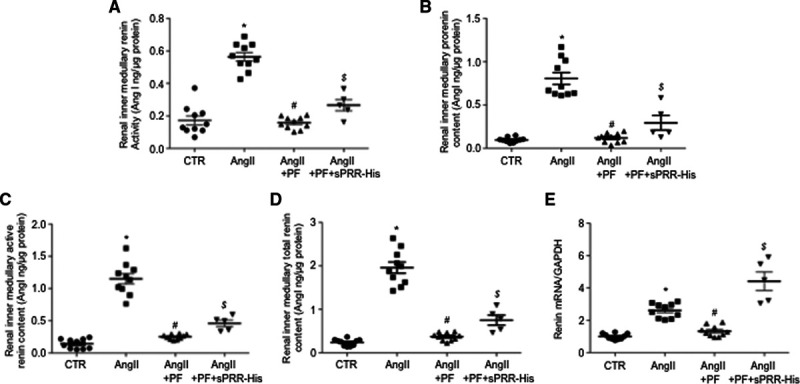
**Assessment of renal inner medullary renin levels and AGT levels in Ang II (angiotensin II) infused mice during S1P (site-1 protease) inhibition alone or supplemented with sPRR (soluble [pro]renin receptor)-His.** B6129SF1/J mice were randomly divided into the following 4 groups: (1) Control (CTR), (2) Ang II, (3) Ang II + PF, and (4) Ang II + PF + sPRR-His. The lysates of the renal inner medulla were assayed for renin activity (**A**), prorenin content (**B**), active renin content (**C**), and total renin concentration (**D**). The values were normalized by protein content. Data are mean ± SE. N=5–10 mice per group. *P<0.05 vs CTR. #*P*<0.05 vs Ang II. $*P*<0.05 vs Ang II + PF. The renal inner medulla was also subjected to quantitative real-time polymerase chain reaction (qRT-PCR) analysis of renin mRNA expression normalized by GAPDH (**E**). Data are mean ± SE. N=5–10 per group. *P<0.05 vs CTR. #*P*<0.05 vs Ang II. $*P*<0.05 vs Ang II + PF.

It has been shown that Ang II–induced intrarenal RAS is reflected by increased urinary renin level paralleled with renal medullary but not renal cortical renin level. Therefore, we detected the inner medullary renin activity, active renin content, prorenin content, and total renin content at day 6 of treatment with control, Ang II, Ang II + PF, or Ang II + PF + sPRR-His. Renin activity, active renin content, prorenin content, and total renin content in the inner medulla were all elevated by Ang II infusion and nearly completely abolished by PF. Addition of sPRR-His infusion partially restored the response of all renin parameters to Ang II in PF treated mice (Figure 3A through 3D). Similar results were obtained by quantitative real-time polymerase chain reaction of renin mRNA (Figure 3E). These results provide further support of S1P-derived sPRR in regulation of local renin response within the renal inner medulla during Ang II infusion.

### Analysis of AGT

A large number of previous studies have shown that urinary angiotensinogen (AGT) excretion reflects activity of intrarenal RAS and predict severity of hypertension^26^ and chronic kidney disease in humans.^27–30^ Accordingly, we have assessed urinary and renal AGT levels by using ELISA and renal AGT mRNA expression by using quantitative real-time polymerase chain reaction. As shown in Figure S2A, urinary AGT excretion was elevated by Ang II infusion, which was blunted by PF and restored by supplement of sPRR-His. Similar patterns of changes were observed for renal AGT mRNA (Figure S2B) and content (Figure S2C). These results provide additional support of regulation of intrarenal RAS by S1P-derived sPRR during Ang II–induced hypertension.

### Analysis of Renal ENaC Expression

**Figure 4.**
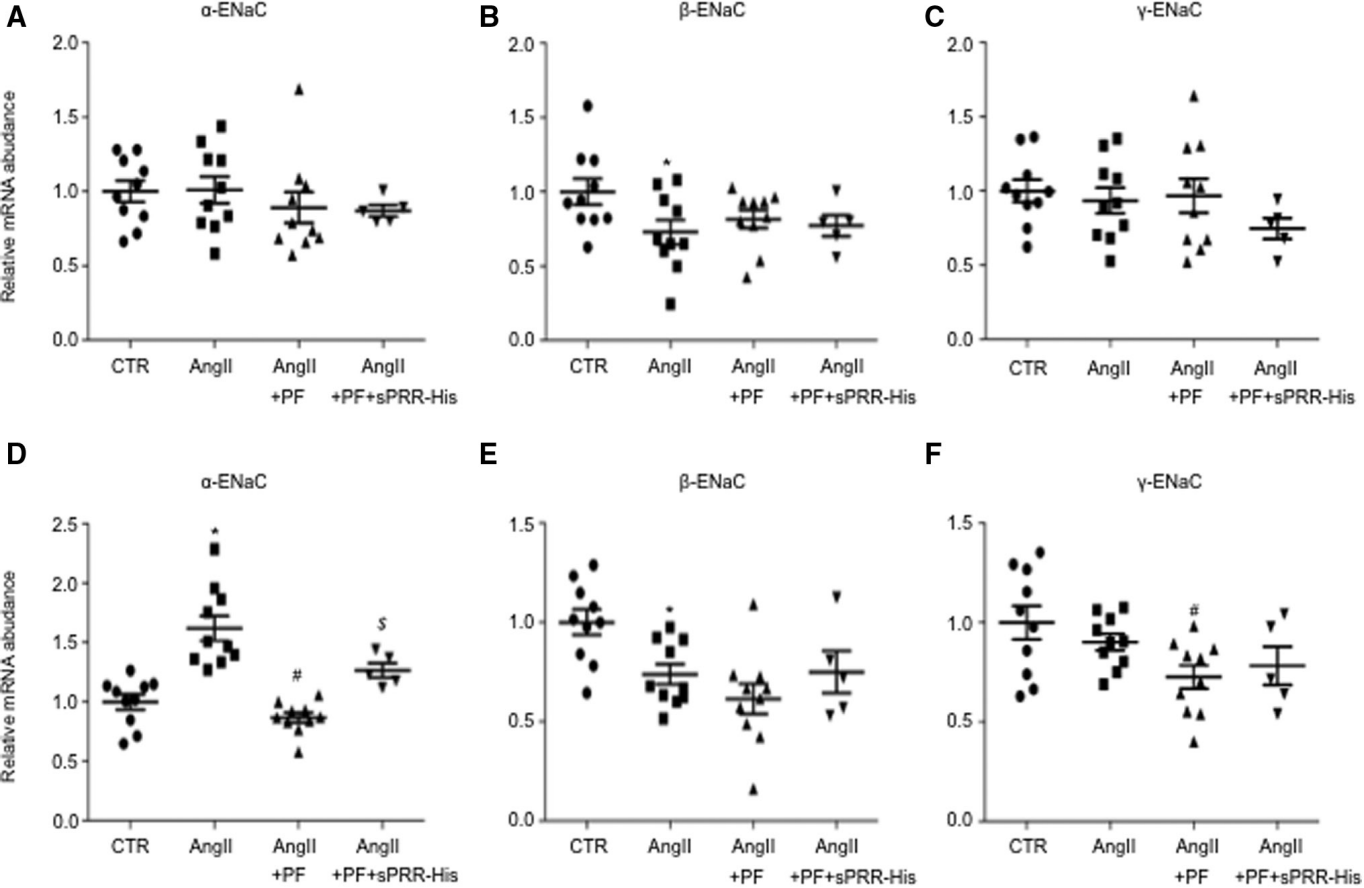
**Assessment of renal regional expression of ENaC subunits in Ang II (angiotensin II) infused mice during S1P (site-1 protease) inhibition alone or supplemented with sPRR (soluble [pro]renin receptor)-His.** B6129SF1/J mice were randomly divided into the following 4 groups: (1) Control (CTR), (2) Ang II, (3) Ang II + PF, and (4) Ang II + PF + sPRR-His. The renal cortex and the renal medulla were subjected to quantitative real-time polymerase chain reaction (qRT-PCR) analysis of mRNA expression of α-ENaC (**Α** and **D**), β-ENaC (**Β** and **E**), and γ-ENaC (**C** and **F**). **A–C**, renal cortex. **D–F**, renal medulla. The mRNA expression was normalized by GAPDH. Data are mean ± SE. N=5–10 mice per group. *P<0.05 vs CTR. #*P*<0.05 vs Ang II. $*P*<0.05 vs Ang II + PF.

**Figure 5.**
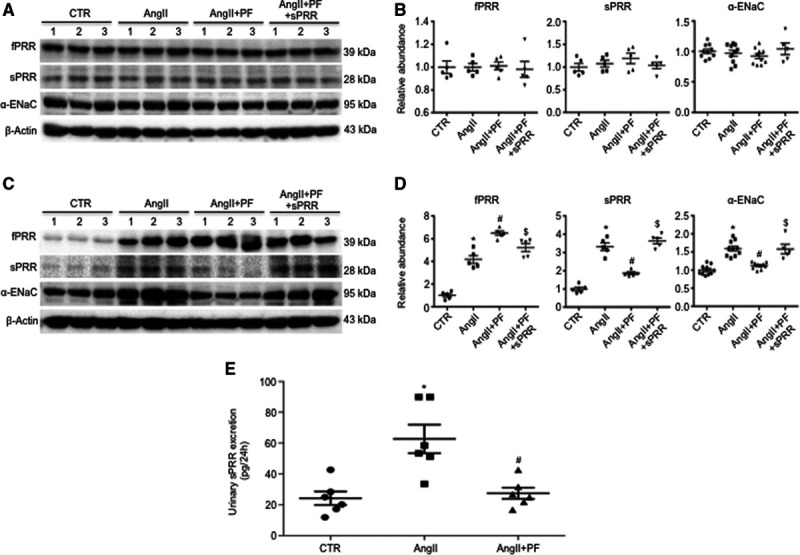
**Assessment of renal protein abundances of α-ENaC and PRR ([pro]renin receptor)/sPRR (soluble PRR), and urinary sPRR excretion in Ang II (angiotensin II) infused mice during S1P (site-1 protease) inhibition alone or supplemented with sPRR-His.** The renal cortex (**A** and **B**) and inner medulla (**C** and **D**) from mice treated with control (CTR), Ang II, Ang II + PF, or Ang II + PF + sPRR-His were subjected to immunoblotting analysis of protein expression α-ENaC and PRR/sPRR. The protein abundances were analyzed by densitometry and normalized by β-actin. The values were shown underneath the blots. Data are mean ± SE. N=5–10 per group. *P<0.05 vs CTR. #*P*<0.05 vs Ang II. $*P*<0.05 vs Ang II + PF. Urine samples were subjected to ELISA measurement of urinary sPRR concertation. The values were expressed as 24 h urine output of sPRR. Data are mean ± SE. N=6 mice per group. *P<0.05 vs CTR. #*P*<0.05 vs Ang II.

We have previously shown that PRR specifically targets renal medullary α-ENaC to mediate the hypertensive response to Ang II.^16,31^ Therefore, we performed quantitative real-time polymerase chain reaction and immunoblotting analysis of the expression of each of the 3 subunits of ENaC in both renal cortex and medulla of mice treated with control, Ang II, Ang II + PF, or Ang II + PF + sPRR-His. Among the 4 groups, renal cortical mRNA expression of all 3 ENaC subunits as assessed by quantitative real-time polymerase chain reaction remained constant (Figure 4A through 4C). In contrast, renal inner medullary α-ENaC mRNA expression was elevated by Ang II, which was blocked by PF and restored by sPRR-His (Figure 4D). Interestingly, renal inner medullary β-ENaC mRNA expression was slightly suppressed by Ang II, which was unaffected by PF or sPRR-His treatment (Figure 4E). However, renal inner medullary γ-ENaC mRNA remained unchanged (Figure 4F). Given the promising changes in renal α-ENaC mRNA expression, subsequent immunoblotting analysis was performed to validate α-ENaC regulation at protein level. While renal cortical α-ENaC protein expression remained constant among the 4 groups (Figure 5A and 5B), renal inner medullary α-ENaC protein expression was upregulated by Ang II, which was suppressed by PF and restored by adding sPRR-His (Figure 5C and 5D). Immunoblotting also demonstrated that Ang II infusion in mice induced a significant increase in sPRR protein abundance in the renal inner medulla but not in the renal cortex; this increase was significantly suppressed in mice by PF and restored by adding sPRR-His (Figure 5A through 5D). Representative Western blot gels for PRR/sPRR and α-ENaC were from 3 animals in each group and the gel results from additional 2 animals in each group were shown as in Figure S3A and S3B. In agreement with this finding, urinary sPRR excretion as determined by ELISA was elevated by Ang II infusion, which was blocked by PF (Figure 5E). These results suggest that S1P-derived sPRR may selectively target renal inner medullary α-ENaC to participate in the hypertension development.

### Analysis of In Vitro ENaC Activity

**Figure 6.**
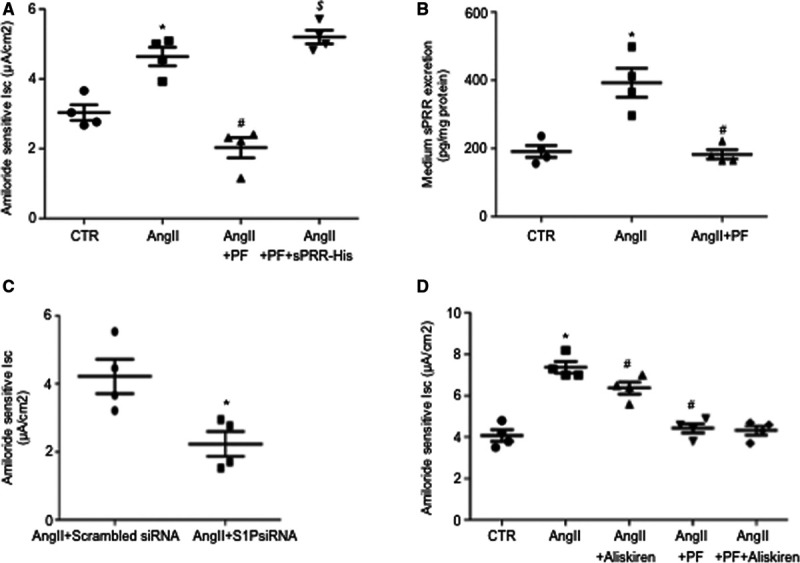
**Role of S1P (site-1 protease)-derived sPRR (soluble [pro]renin receptor) in mediation of Ang II (angiotensin II)–induced ENaC activation in vitro.** Confluent mouse cortical collecting duct cell line (mpkCCD) cells grown on Snapwells were pretreated with 10 μmol/L PF for 1 h and then treated with 1 μmol/L Ang II and 10 nmol/L sPRR-His for 24 h. Amiloride-sensitive transepithelial Na^+^ transport, an index of ENaC activity, was recorded by the Ussing chamber technique. Medium sPRR was measured by using ELISA. **A**, ENaC activity in CTR, Ang II, Ang II + PF, and Ang II + PF + sPRR-His groups. Data are mean ± SE. N=4 per group. *P<0.05 vs CTR. #*P*<0.05 vs Ang II. $*P*<0.05 vs Ang II + PF. **B**, Medium sPRR content in cells treated with CTR, Ang II, or Ang II + PF were determined by using ELISA. Data are mean ± SE. N=4 per group. *P<0.05 vs CTR. #*P*<0.05 vs Ang II. **C**, The cells were transfected with S1P siRNA or scrambled siRNA, followed by 24-h Ang II treatment and Ussing chamber measurement of ENaC activity. Date are mean ± SE. N=4 per group. *P<0.05 vs scrambled siRNA. **D**, The cells were pretreated with 1 μmol/L Aliskiren for 0.5 h or 10 μmol/L PF for 1 h, followed by 24-h Ang II treatment and Ussing chamber measurement of ENaC activity. Date are mean ± SE. N=4 per group. *P<0.05 vs CTR. #*P*<0.05 vs Ang II.

The in vitro electrophysiology study was conducted to examine the direct role of S1P-derived sPRR in regulation of ENaC activity in the setting of Ang II treatment. Confluent mpkCCD cells were grown on Transwell membrane and exposed for 24 hours to Ang II, Ang II + PF, or Ang II + PF + sPRR-His followed by Ussing chamber measurement of amiloride-sensitive short-circuit current, an index of ENaC activity. Ang II–induced ENaC activity was blocked by PF, which was reversed by adding sPRR-His (Figure 6A). siRNA-mediated knockdown of S1P similarly attenuated Ang II–induced ENaC activity (Figure 6B). Furthermore, medium sPRR as measured by ELISA was elevated by Ang II, which was blunted by PF (Figure 6C).

Renin activity along with PRR expression is elevated in cultured CD cells exposed to Ang II. We sought to determine the involvement of renin activity versus PRR in mediating Ang II–induced ENaC activity in cultured mpkCCD cells by using the Ussing chamber technique. We found that the renin inhibitor Aliskiren treatment induced a small but significant attenuation of Ang II–induced ENaC activity (Figure 6D). Relatively, the S1P inhibition with PF was much more efficient than Aliskiren and combination of the 2 inhibitors revealed no additive effect (Figure 6D). These results support a predominant role of S1P-derived sPRR in mediating Ang II–induced ENaC activity, whereas the involvement of renin activity may be minimal.

## Discussion

The present study for the first time examined the role and mechanism of sPRR derived from S1P, a newly discovered PRR cleavage protease, during Ang II–induced hypertension. We found that pharmacological inhibition of S1P effectively attenuated Ang II–induced hypertension, and this effect was reversed by supplement with exogenous sPPR-His. Further evidence supports that sPRR promotes hypertension development by enhancement of intrarenal RAS and activation of renal inner medullary α-ENaC.

Recently, a series of studies from our group and others have begun to unravel the biological function of sPRR^32,33^ as well as identity of the cleavage proteases.^5,6^ The present study is the first to show that S1P-derived sPRR contributes to Ang II–induced hypertension. This conclusion is based on a series of data sets. First, renal medullary but not renal cortical protein abundance of sPRR was increased by Ang II infusion, which was reversed by PF, indicating site-specific enhancement of sPRR generation in the renal medulla via the activity of S1P. In support of this notion, the increase of renal PRR protein expression in response to Ang II infusion took place selectively in the renal medulla but not renal cortex, providing the substrate for renal medullary generation of sPRR. Second, S1P inhibition effectively attenuated Ang II-induced increases in MAP, SBP and DBP. Third, the rescue experiment with the use of exogenous sPRR-His supports the causative role of S1P-derived sPRR in mediating Ang II–induced hypertension. Besides PRR, S1P modifies unique membrane-bound latent transcription factors. A well-studied representative of this type of transcription factors is the SREBP (sterol regulatory element-binding transcription factor), a crucial transcription factor governing cholesterol and fatty acid biosynthesis.^34–37^ Similarly, S1P also processes membrane-bound ATF6 (activating transcription factor 6) during ER stress response.^38^ Currently, no evidence is available to suggest involvement of SREBP or ATF6 in regulation of Ang II–induced hypertension.

Existence of the intrarenal RAS is highlighted by the expression of renin in the CD,^39–41^ where the expression is increased by Ang II infusion,^42,43^ contrasting to suppressed plasma renin. In cultured CD cells, Ang II directly stimulates renin expression.^43^ Overactivation of intrarenal RAS contributes to Ang II–induced hypertension.^22,23,25,44^ A series of studies from our group demonstrate dependence of Ang II–induced intrarenal renin on PRR. For example, intramedullary infusion of a PRR decoy inhibitor PRO20 in uninephrectomized rats suppressed intrarenal renin level that paralleled with a BP-lowering effect.^12^ Furthermore, conditional deletion of PRR from the CD largely recapitulated the effects of PRO20 during Ang II infusion.^16^ In extension of these observations, the present study demonstrates that the renin regulatory role of PRR is mediated by S1P-dependent generation of sPRR. Additionally, urinary AGT excretion, a well-recognized index of intrarenal RAS, was also under the control of S1P-derived sPRR in the current experimental model. Furthermore, urinary AGT excretion correlated well with renal AGT content and mRNA expression, suggesting renal generation of AGT. This finding is important in light of conflicting reports in the literature concerning renal^45^ versus hepatic^45,46^ origin of AGT as a major substrate for renal Ang II production.

The mechanism of how sPRR regulates intrarenal renin remains elusive. Like PRR, sPRR is capable of binding prorenin and renin to increase their catalytic activity presumably through the conformational change of the substrates.^1^ It is interesting to note that besides renin activity, active renin content, prorenin content, and total renin content in the current Ang II infusion model were all affected by S1P inhibition and further restored by sPRR supplement. The broad suppression of multiple renin parameters was also seen following intramedullary infusion of PRO20 in rats^12^ or CD-specific deletion of PRR.^16^ These results may raise a possibility that sPRR-dependent regulation of renin may occur at multiple levels in addition to catalytic activity. Future studies are needed to examine if sPRR regulates prorenin/renin content at the level of gene expression or protein stability.

Along the CD, most Na^+^ transport is thought to occur through ENaC in the CCD.^47^ In contrast, Na^+^ transport in the medullary part of CD is often neglected. It is intriguing that expression of α-ENaC but not β- or γ-subunit was elevated in the renal inner medulla but not renal cortex of floxed mice following Ang II infusion, and this elevation was completely blocked by CD PRR deletion.^16^ These results suggest that PRR-dependent activation of ENaC-mediated Na^+^ reabsorption in the terminal nephron segment may in part contribute to Ang II–induced hypertension. This result is in agreement with the observation that PRR knockdown by a shRNA-based approach selectively reduced α-ENaC subunits in the renal medulla, although PRR is suppressed in the whole kidney.^48^ More recently, we report that in primary rat inner medullary collecting duct (IMCD) cells, administration of sPRR-His at 10 nmol/L for 24 hours induced protein expression of α- but not β- or γ-subunit of ENaC, in parallel with upregulation of mRNA expression as well as promoter activity of the α-subunit. The transcriptional activation of α-ENaC was dependent on β-catenin signaling.^31^ In agreement with these observations, the present study provided additional evidence for a role of S1P-derived sPRR in mediating Ang II–induced selective upregulation of renal medullary expression of α-ENaC. Along this line, the in vitro electrophysiological studies demonstrate the direct role of S1P-derived sPRR in mediating Ang II-induced activation of ENaC activity in cultured CD cells. The regional differences in Ang II–induced α-ENaC expression agrees well with the observation that protein abundance PRR and sPRR induced by Ang II infusion occurred only in the renal medulla but not renal cortex. These results suggest a novel sPRR/α-ENaC pathway in regulation of renal medullary function and BP and support our overall hypothesis that S1P-derived sPRR targets renal inner medullary α-ENaC to stimulate tubular Na^+^ reabsorption leading to elevated BP. However, it is important to note that the restricted upregulation of renal inner medullary α-ENaC during Ang II–induced hypertension may disagree with other report that all 3 ENaC subunits were similarly induced in both renal cortex and medulla in this model.^49^ The reason for this discrepancy is unclear at this moment but may be related to differences in experimental protocols or animal species (mouse versus rat).

As a key feature of intrarenal RAS, the expression of renin in the CD is upregulated by Ang II contrasting to the negative feedback regulation of renin release from the juxtaglomerular apparatus. Although this phenomenon is well established, its functional implication is unclear. In the present study, we used renin inhibitor Aliskiren to examine the role of renin activity in ENaC regulation by Ang II. We found that Aliskiren only induced a modest attenuation of Ang II–induced ENaC activity contrasting to the more complete inhibition with PF. These results support the concept that sPRR may regulate ENaC activity largely independent of renin activity.

## Perspectives

The prevalence of hypertension is increasing with nearly 1 out of 2 adults in the United States suffering from this disease and the BP being uncontrolled for most of the cases.^50^ It is of high significance to understand the disease mechanism as well as the therapeutic target. A large body of experimental evidence has established PRR as a key player in the pathogenesis of hypertension induced by a variety of stimuli such as Ang II infusion or fructose/salt treatment.^51^ Recent work demonstrates S1P as a predominant source of sPRR. The present study is the first to report an essential role of S1P/sPRR pathway in mediating Ang II–induced hypertensive response in mice. This pathway may offer a new therapeutic target for management of hypertension in patients with elevated Ang II levels. S1P/sPRR pathway seems to target intrarenal RAS and renal inner medullary α-ENaC to increase BP. However, the mechanism immediately downstream of sPRR, such as the direct receptor for sPRR, remains to be determined in future studies.

## Affiliation

## Sources of Funding

This work was supported by the National Institutes of Health Grants HL139689, DK104072, HL135851, and VA Merit Review from the Department of Veterans Affairs. T. Yang is a Research Career Scientist in Department of Veterans Affairs.

## Disclosures

None.

## Supplementary Material



## Novelty and Significance

### What Is New?

- Recent evidence has established S1P (site-1 protease) as a predominant source of sPRR (soluble [pro]renin receptor).
- The current study is the first to assess the role of S1P-derived sPRR in pathogenesis of Ang II (angiotensin II)–induced hypertension.

### What Is Relevant?

- RAS inhibition represents a major therapeutic strategy for management of hypertension and its associated cardiovascular and renal disease, highlighting an essential role of Ang II in these diseases.
- The current work contributes to defining S1P/sPRR pathway as a new mechanism of Ang II–induced hypertension.

### Summary

We, for the first time, demonstrated an essential role of S1P-derived sPRR in mediating the hypertensive response to Ang II infusion in mice. Moreover, we provided a mechanism of this phenomenon, which involves the activation of intrarenal RAS and selective stimulation of renal medullary α-ENaC expression. In vitro evidence further supported a direct role of S1P-derived sPRR in regulation of ENaC activity during Ang II treatment. The S1P/sPRR pathway may provide a novel target for antihypertensive therapy.
