# Supplementary material for: Site-1 Protease-Derived Soluble (Pro)Renin Receptor Contributes to Angiotensin II–Induced Hypertension in Mice
Source: Hypertension. 2020 Dec 7;77(2):405–16. doi: 10.1161/HYPERTENSIONAHA.120.15100 (PMC7803453; doi:10.1161/HYPERTENSIONAHA.120.15100)

Online Supplement

Site-1 Protease-Derived Soluble (Pro)Renin Receptor Contributes to Angiotensin II-Induced Hypertension in Mice

Ye Feng^1^, Kexin Peng^1^, Renfei Luo^1^, Fei Wang^1^, and Tianxin Yang^1^

^1^Department of Internal Medicine, University of Utah and Veterans Affairs Medical Center, Salt Lake City, Utah, USA.

Running title: sPRR and AngII hypertension

Address correspondence to:

Tianxin Yang, M.D., Ph.D.

University of Utah and Veterans Affairs Medical Center

Division of Nephrology and Hypertension

30N 1900E, RM 4C224

Salt Lake City, UT 84132

Tel: 801-585-5570

Fax: 801-584-5658

Email: [Tianxin.Yang@hsc.utah.edu](mailto:Tianxin.Yang@hsc.utah.edu)

**Methods**

*Mouse experiments***.** B6129SF1/J mice were infused for 6 days with control (CTR), AngII (AngII at 300 ng/kg/day), AngII + PF (PF at 20 mg/kg/day) (HY-13447A, Medchem Express) via a subcutaneously implanted minipump (Alzet model 1007D, Alza) or in conjunction with i.v.sPRR-His infusion at 30 μg/kg/day via jugular vein catheterization connected to a separate osmotic minipump as previously reported [^15^](#_ENREF_15). The same doses of AngII, PF, and sPRR-His have been validated by our previous studies [^15^](#_ENREF_15)^,^ [^16^](#_ENREF_16). The radiotelemetric device was implanted via catheterization of carotid artery and was turned on for 4 h per day from 5:00 PM to 9:00 PM. At the end of the experiment, under general anesthesia, urine was collected from puncturing bladder, blood was withdrawn by puncturing vena cava, and the kidney was harvested and cut into cortex and the inner medulla and snaps frozen. Twenty-four hours urine collection was performed on separate groups of the animals receiving the same treatments except that they were not instrumented with radiotelemetric devices.

*Immunobloting*. Renal tissues including the cortex and the inner medulla were lysed and subsequently sonicated in PBS that contained 1% Triton x-100, 250 μM phenylmethanesulfonyl fluoride (PMSF), 2 mM EDTA, and 5 mM dithiothrietol (DTT) (pH 7.5). Protein concentrations were determined by the use of Coomassie reagent. 40 μg of protein for each sample was denatured in boiling water for 10 min, then separated by SDS-PAGE, and transferred onto nitrocellulose membranes. The blots were blocked overnight with 5% nonfat dry milk in Tris-buffered saline (TBS), followed by incubation for overnight with primary antibody. After being washed with TBS, blots were incubated with horseradish peroxidase (HRP)-conjugated secondary antibody and visualized using Enhanced Chemiluminescence (ECL). The blots were quantitated by using Imagepro-plus. Primary antibodies are as follows: rabbit anti-PRR antibody (cat no. HPA003156; Sigma), rabbit anti-α-ENaC antibody (cat no. SPC-403D; Stressmarq Biosciences), mouse anti-β-actin antibody (cat no. A1978; Sigma).

*Enzyme immunoassay*. Angiotensinogen (AGT) in urine and kidney were determined by using the following commercially available enzyme immunoassay (EIA) according to the manufacturer’s instructions: the kit for AGT (Cat no. MBS268355, BioSource).

Figure legend

Figure S1. Body weights in AngII infused mice during S1P inhibition alone or supplemented with sPRR-His. B6129SF1/J mice were randomly divided into the following four groups: 1) CTR, 2) AngII, 3) AngII + PF, and 4) AngII + PF + sPRR-His.


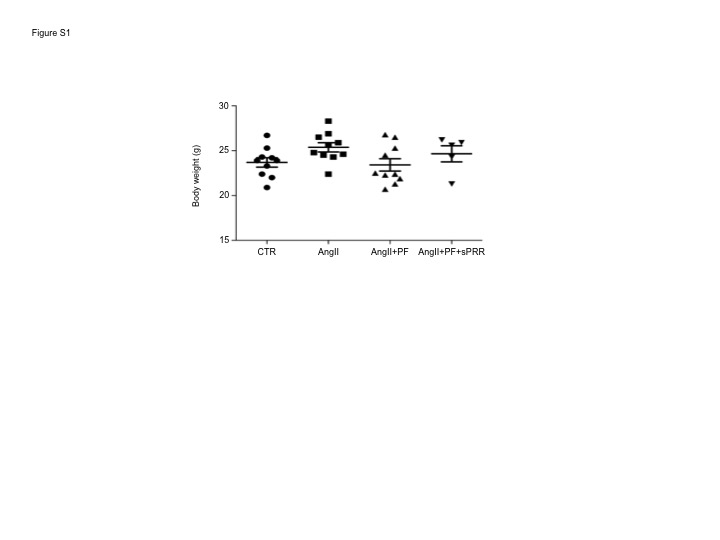


Figure S2**.** Assessment of renal AGT levels in AngII infused mice during S1P inhibition alone or supplemented with sPRR-His. B6129SF1/J mice were randomly divided into the following four groups: 1) CTR, 2) AngII, 3) AngII + PF, and 4) AngII + PF + sPRR-His. The kidney was subjected to qRT-PCR analysis of mRNA expression of AGT (B). The mRNA expression was normalized by GAPDH. Data are mean ± SE. N = 5 mice per group. *, p <0.05 vs. CTR. #, p <0.05 vs. AngII. $, p <0.05 vs. AngII + PF. Urinary AGT excretion (A) and renal AGT (C) were determined by using ELISA. Data are mean ± SE. N = 4-5 mice per group. *, p <0.05 vs. CTR. #, p <0.05 vs. AngII. $, p <0.05 vs. AngII + PF.


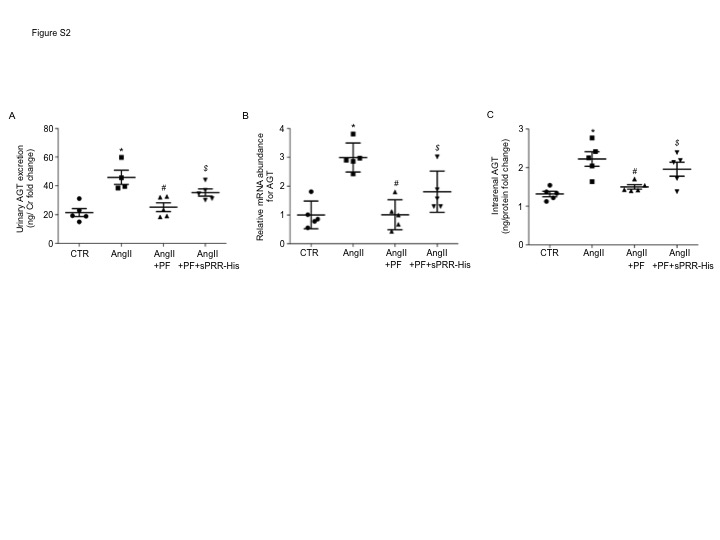


Figure S3**.** Assessment of renal protein abundances of α-ENaC and PRR/sPRR in AngII infused mice during S1P inhibition alone or supplemented with sPRR-His. The results were obtained from two series of experiments. The animal in each group has been numbered. A, C, and D: renal cortex; B, E, and F: renal inner medulla. The representative Western blot gel results from the first 3 animals (#1-3) have been shown in Fig. 5.


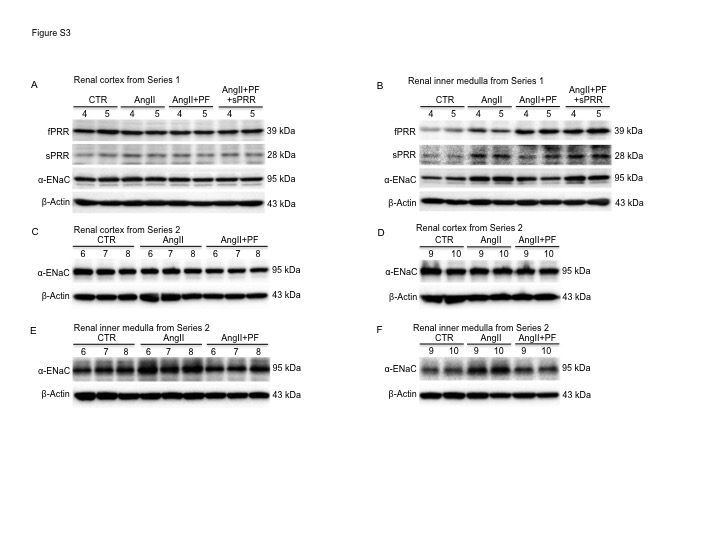

Supplement: Supplementary file 1 [file hyp-77-405-s001.docx]
