# Supplementary figures and images for: Site-1 Protease-Derived Soluble (Pro)Renin Receptor Contributes to Angiotensin II–Induced Hypertension in Mice
Source: Hypertension. 2020 Dec 7;77(2):405–16. doi: 10.1161/HYPERTENSIONAHA.120.15100 (PMC7803453; doi:10.1161/HYPERTENSIONAHA.120.15100)

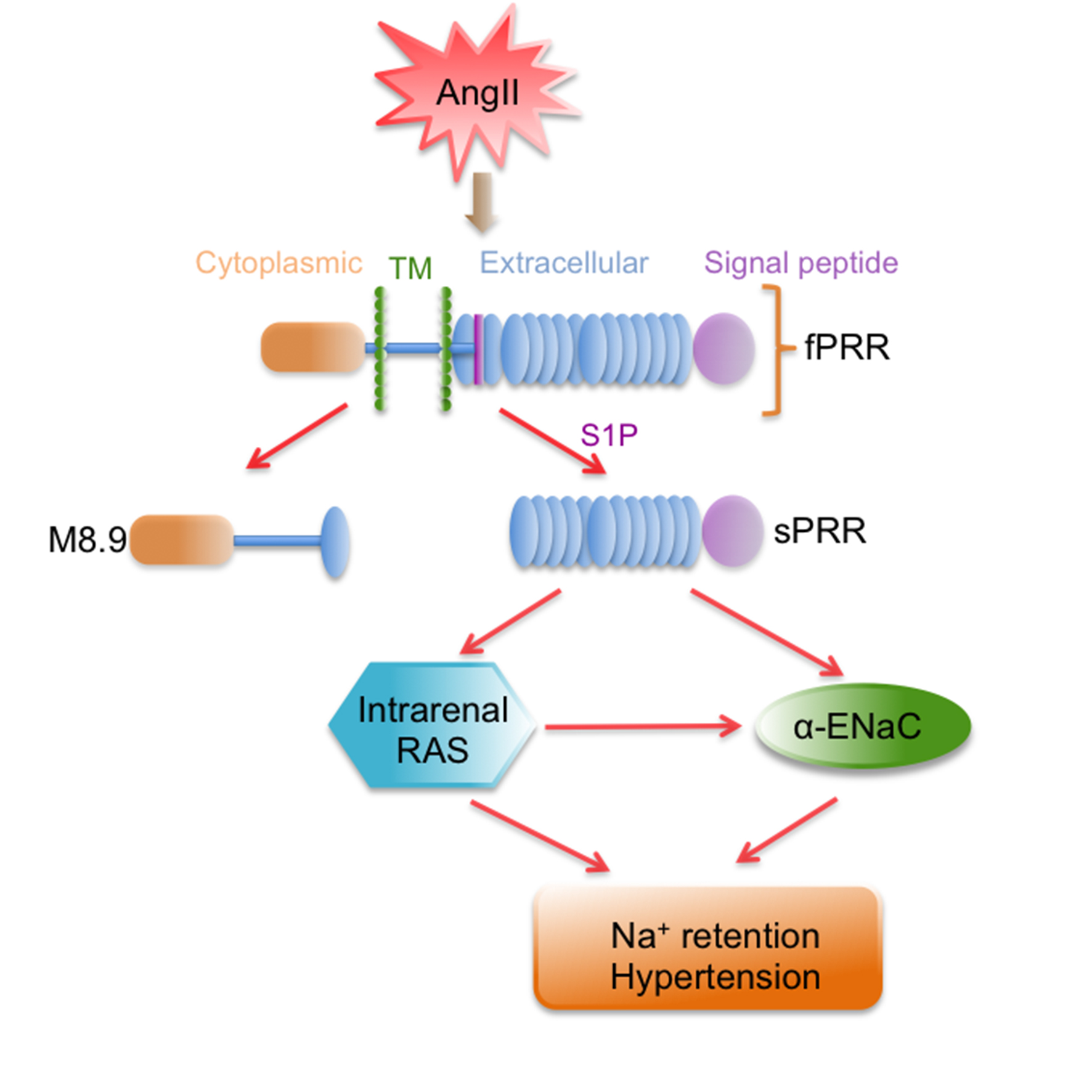

Supplement: Supplementary file 3 [file hyp-77-405-s003.jpg]
