# Supplementary material for: Site-1 Protease-Derived Soluble (Pro)Renin Receptor Contributes to Angiotensin II–Induced Hypertension in Mice
Source: Hypertension. 2020 Dec 7;77(2):405–16. doi: 10.1161/HYPERTENSIONAHA.120.15100 (PMC7803453; doi:10.1161/HYPERTENSIONAHA.120.15100)
Supplement: Supplementary file 4 [file hyp-77-405-s004.pdf]

*** Short In Vivo Checklist**
AHA - Preclinical Animal Testing: Prevention of bias is important for experimental cardiovascular research. ***This short checklist must be completed, and the answers should be clearly presented in the manuscript as well.*** *The checklist will be used by reviewers and editors but will not be published. If a revision is requested, you will be required to complete at revision submission a more detailed checklist that will be published with the accepted article.*

This study involves animals:
Yes

Animals

Species, age, sex, strains, and sources of animals are described: Yes

Randomization

Randomization and allocation concealment were performed: Yes

Blinding

Blinding was performed: N/A

Inclusions and Exclusions (a)

Specific criteria for inclusions and exclusions are specified: N/A

Inclusions and Exclusions (b)

Criteria for inclusions and exclusions were set before the study: N/A

Reporting of Excluded Animals

All animals excluded after the randomization are reported: N/A

Statistical Methods

Statistical Methods are described: Yes

Date completed: 09/29/2020 16:53:27
User pid: 11987
